# Editorial: Complexity Characteristics of Natural Language

**DOI:** 10.3390/e28010098

**Published:** 2026-01-14

**Authors:** Stanisław Drożdż, Jarosław Kwapień, Tomasz Stanisz

**Affiliations:** 1 Complex Systems Theory Department, Institute of Nuclear Physics, Polish Academy of Sciences, 31-342 Kraków, Poland; 2 Faculty of Computer Science and Mathematics, Cracow University of Technology, 31-155 Kraków, Poland

The contributions collected in this Special Issue testify to the continued vitality and conceptual breadth of research at the intersection of natural language, complexity science, and information theory. Taken together, the ten papers presented here advance a shared perspective that natural language is not merely a vehicle for meaning, but rather a multiscale complex system whose statistical, dynamical, and structural properties encode deep regularities of human cognition, social interaction, and cultural evolution.

Several contributions deepen our quantitative understanding of scaling, correlations, and variability in language. Detailed analyses of punctuation and sentence-length variability reveal that punctuation is not a superficial stylistic device but rather a key organizer of linguistic correlations, capable of inducing multifractal structure and long-range dependence in texts. Studies of experimental literary works demonstrate that even highly unconventional narratives retain or sometimes even amplify these complexity signatures, and in some cases show remarkable translation invariance, pointing to constraints that transcend individual languages.

Other papers broaden the empirical scope of linguistic complexity by examining language across spatial, temporal, grammatical, and genealogical scales. Large-scale analyses of social media data show how rank diversity and scaling laws depend jointly on grammar, geography, and time, revealing both universal patterns and context-sensitive deviations. At a macroscopic level, the study of language families uncovers Zipf-like regularities that may reflect long-term “entropic” processes of diversification and contact, connecting linguistic evolution to ideas from statistical physics.

A third thematic strand addresses causality and structure in linguistic systems. Some contributions move beyond correlation by explicitly asking what causes what in language change and use. By combining information-theoretic complexity measures with causal inference techniques, evidence is provided demonstrating that changes in morphological complexity can precede and drive changes in syntactic organization, rather than the reverse. Complementary work critically reassesses long-standing hypotheses about social drivers of complexity, showing—using improved data and modeling—that the proportion of non-native speakers does not straightforwardly reduce linguistic complexity.

This Special Issue also connects classical complexity questions with contemporary NLP and machine learning. Some contributions explicitly engage with large language models (LLMs), either as analytical tools or as objects of study. Novel approaches combine linguistic structure (e.g., relational triples) with LLMs to address tasks such as objectivity detection in informal texts. Other contributions show that, despite their impressive fluency, current LLM-generated texts lack certain hallmark properties of natural language—most notably robust long-range dependence—highlighting a gap between human language production and present-day architectures. Work on informativity and predictability further clarifies how information-theoretic concepts should be interpreted when applied to noisy corpus data and probabilistic models, while a forward-looking analysis discusses both the promise and the limitations of LLMs for causal reasoning in text.

Despite decades of progress, several gaps have persisted in the study of language complexity. In particular, many earlier studies relied on static correlations, leaving causal relationships underspecified. Results were often fragmented across linguistic levels (characters, words, syntax, discourse) and data types (literary texts, spoken language, social media). The rapid rise of LLMs created an urgent need to reassess classical complexity measures in light of generative models that can mimic surface statistics without penetrating deeper dependencies. This Special Issue directly addresses these gaps by (i) integrating causal inference with information-theoretic and algorithmic complexity measures, (ii) systematically comparing multiple linguistic scales and data sources, and (iii) explicitly contrasting human-generated and machine-generated language. In doing so, the collected works show that complexity is not a monolithic property but an emergent outcome of interacting constraints operating across time, scale, and representation.

Looking ahead, the interaction between complexity science and large language models is likely to define the next phase of research. Several directions stand out as particularly suggestive. Among these, empirical evidence that natural language exhibits long-range dependence and multifractal organization indicates that future LLM architectures must incorporate richer, more persistent memory mechanisms. Understanding which complexity signatures matter most for meaning, coherence, and creativity remains an open challenge. As models become capable of producing fluent explanations, distinguishing genuine causal understanding from plausible-sounding narratives will be of crucial importance. Complexity-based diagnostics may help us to evaluate whether LLMs capture causal structure or merely approximate correlations. Extending complexity analyses to low-resource languages, multimodal communication, and hybrid human–AI texts will test the universality of observed laws and reveal where current models fall short. Rather than treating LLMs as black boxes, future work should use insights from information theory, scaling laws, and algorithmic complexity to design principled benchmarks that go beyond accuracy and perplexity.

In conclusion, this Special Issue demonstrates that the study of natural language complexity remains both theoretically rich and practically relevant. By combining rigorous quantitative methods with emerging AI technologies, the field is well positioned to deepen our understanding of language as a complex adaptive system and to guide the development of language models that more faithfully reflect its structure and dynamics.

